# Diagnostic Value of Autoantibodies against Steroidogenic Enzymes and Hormones in Infertile Women with Premature Ovarian Insufficiency

**DOI:** 10.3390/ijms25126545

**Published:** 2024-06-14

**Authors:** Leila V. Adamyan, Irina V. Menzhinskaya, Alena A. Antonova, Narine M. Tonoyan, Gennady T. Sukhikh

**Affiliations:** 1 National Medical Research Center for Obstetrics, Gynecology and Perinatology Named after Academician V.I. Kulakov of the Ministry of Health of the Russian Federation, 117997 Moscow, Russia; 2 Department of Obstetrics, Gynecology and Reproductive Medicine, Federal State Budgetary Educational Institution of Higher Education “Russian University of Medicine” of the Ministry of Health of the Russian Federation, 127473 Moscow, Russia; 3 Department of Obstetrics, Gynecology, Perinatology and Reproductology, Institute of Professional Education, I.M. Sechenov First Moscow State Medical University of the Ministry of Health of the Russian Federation (Sechenov University), 119991 Moscow, Russia

**Keywords:** premature ovarian insufficiency, infertility, diminished ovarian reserve, autoimmunity, autoantibodies against steroidogenic enzymes, cholesterol side-chain cleavage enzyme, aromatase, 21-hydroxylase

## Abstract

The objective of the study was to evaluate the profile and diagnostic significance of serum autoantibodies in infertile patients with premature ovarian insufficiency (POI). The pilot study included 26 patients of reproductive age with POI and diminished ovarian reserve who received complex treatment using new surgical technologies (Group 1) and 18 patients without POI (Group 2). The profile of serum autoantibodies, including anti-ovarian antibodies, antibodies against thyroid peroxidase (TPO), steroidogenic enzymes, and steroid and gonadotropic hormones, was studied using modified ELISAs and human recombinant steroidogenic enzymes (CYP11A1, CYP19A1, CYP21A2). Patients in Group 1 had higher levels of IgG autoantibodies against steroidogenic enzymes, estradiol, progesterone, and TPO than those in Group 2. Tests for IgG antibodies against CYP11A1, CYP19A1, and CYP21A2 exhibited high sensitivity (65.4–76.9%), specificity (83.3–89.9%), and AUC values (0.842–0.910) for POI, the highest in the first test. Three-antibodies panel screening showed higher diagnostic accuracy (84.1% versus 75–79.6%). The levels of these antibodies correlated with menstrual irregularities and a decrease in the antral follicle count. Thus, antibodies against CYP11A1, CYP19A1, and CYP21A2 have a high diagnostic value for POI. Three-antibody panel screening may improve the accuracy of POI diagnosis and be useful for identifying high-risk groups, early stages of the disease, and predicting POI progression.

## 1. Introduction

Premature ovarian insufficiency (POI) is a serious problem in reproductive medicine and is a clinical syndrome defined by the loss of ovarian function in women before the age of 40 years [1,2,3]. POI has a detrimental effect on women’s physical and psychological health, as well as their quality of life [3]. The overall prevalence of POI in the world today is 3.5%. In developed countries, it is 3.1%. In developing countries, it is higher, reaching 5.3% [4]. It is important to note that there has been a tendency towards an increase in the prevalence of POI over the last 20 years [4]. According to one study, the risk of POI among relatives of women with POI is 4.6 times higher than among relatives of women without POI [5].

POI is characterized by a decrease in the level of sex steroid hormones; loss of residual follicles; menstrual abnormalities, such as oligomenorrhea and amenorrhea; and infertility [3,6]. The diagnosis of POI is based on the European Society of Human Reproduction and Embryology (ESHRE) criteria, which include the following: amenorrhea or oligomenorrhea for at least four months and elevated serum follicle-stimulating hormone (FSH) levels greater than 25 IU/L, measured twice at least four weeks apart [7]. However, at the time of diagnosis of POI, many women may have normal anti-Müllerian hormone (AMH) levels and a preserved pool of residual ovarian follicles that decreases over time [8].

Clinical symptoms of POI caused by both estrogen and androgen deficiency include vulvovaginal atrophy, low libido, dyspareunia, changes in urinary frequency and recurrent infections, vasomotor instability, sleep disturbances, emotional lability, and depression [3]. In this regard, patients with POI frequently require psychological counseling [9]. A premature reduction in estrogen increases the risk of cardiovascular disease, parkinsonism, osteoporosis, hypertension, weight gain, midlife diabetes, and cognitive disorders and dementia, such as Alzheimer’s disease (AD) [10].

The development of POI is caused by the action of various etiological factors, namely: genetic, autoimmune, iatrogenic, and environmental factors. However, there is a high incidence of cases of idiopathic POI (74–90%), in which the cause of the disease remains unclear [11], which may be due to insufficient diagnostic efficiency. The pathophysiology of this condition remains under investigation with limited data in humans [8].

Autoimmune genesis is detected in 4–30% of cases of POI [1,4,12] and is confirmed by the presence of anti-ovarian antibodies (AOA) or histological signs of lymphocytic oophoritis or concomitant autoimmune diseases [13]. In this case, steroid-producing cells in pre-ovulatory follicles and the corpus luteum are the main targets of autoantibodies in the ovaries. Consequently, autoimmune attacks can lead to the destruction of follicles, a decrease in their number, and fibrosis of the ovarian stroma [1].

Patients with POI are often susceptible to autoimmune diseases. POI is frequently associated with autoimmune diseases of the thyroid gland (Hashimoto’s thyroiditis, Graves’ disease) in 14–32.7% of cases and adrenal glands (Addison’s disease or primary adrenal insufficiency) in 10–20% of cases [3] and is also detected in combination with other autoimmune diseases such as rheumatoid arthritis, Crohn’s disease, myasthenia gravis, systemic lupus erythematosus, and multiple sclerosis [3,14].

Lymphocytic oophoritis often precedes the development of POI, especially when combined with Addison’s disease. It is also closely linked to autoimmune polyglandular syndrome (APS) [14]. However, histological confirmation is only achieved in 10% of cases of POI due to the necessity for ovarian biopsy [12,15]. The difficulties in establishing a diagnosis are due to the transient nature of oophoritis and the biphasic nature of POI, manifested by atrophy only at the final stage of the disease [5,16,17]. Autoimmune oophoritis is characterized by impaired cellular and humoral immunity and is associated with the presence of autoantibodies to steroidogenic enzymes of cytochrome P450, such as 21-hydroxylase (21OH, P450c21), 17α-hydroxylase (17αOH, P450c17), cholesterol side-chain cleavage enzyme (P450scc), and also to 3β-hydroxysteroid dehydrogenase (3β)-HSD) [15].

Given the association between POI and autoimmune diseases of the adrenal gland and thyroid gland, ESHRE recommends routine screening for both anti-thyroid peroxidase (anti-TPO) antibodies and anti-21OH, or adrenocortical autoantibodies, in every case of POI [7]. The immunofluorescence test, or immunoprecipitation method with ligands labeled with a radioisotope (35S-methionine or iodine-125 (125I)) or luminescent (luciferase) label, is the most commonly employed method for the detection of these antibodies [3]. Recently, an enzyme-linked immunosorbent assay (ELISA) was proposed as a means of detecting antibodies to 21OH [18]. Although antibodies to 21OH are a laboratory diagnostic criterion for Addison’s disease, these antibodies are detected in patients with POI in 30% of cases long before the manifestation of adrenal disease [1,19,20]. 

Currently, the diagnosis of autoimmune POI represents a significant challenge because there are no established diagnostic criteria and no antibody tests with high specificity for autoimmune POI. Additionally, ovarian biopsy is of limited use due to the risk of injury to residual follicles [8]. In this regard, the search for specific markers of POI continues in order to create effective methods for the early diagnosis and prognosis of the disease, which are available for laboratory practice.

In light of the aforementioned considerations, the objective of this study was to evaluate the profile and diagnostic significance of serum autoantibodies in infertile patients with premature ovarian insufficiency.

## 2. Results

All women in the study groups were of reproductive age (18–45 years) and were matched in age and body mass index (BMI) (Table 1). According to the family history of patients with POI, the median age at menopause among their relatives was 52 (35–55) years. However, in 15.4% of cases (4/26) the age of menopause was less than 45 years.

All patients with POI complained mainly of the absence of pregnancy and menstruation or irregular menstrual cycle, as well as hot flashes, sweating, decreased libido, insomnia, and anxiety. In 34.6% of patients (9/26), the effect of stress factors preceded the onset of clinical manifestations of POI. Patients had menstrual irregularities for 1 (0–12) years: secondary amenorrhea (in 69.2% of cases (18/26)), oligomenorrhea (in 30.8% of cases (8/26)). Infertility was observed in 100% of cases, while primary infertility was diagnosed more often (in 65.4% of patients (17/26)) than secondary infertility (in 34.6% of patients (9/26); *p* = 0.028). Moreover, primary infertility was detected more often in Group 1 than in Group 2 (22.2%; *p* = 0.0053). Previous failed in vitro fertilization (IVF) cycles using own or donor oocytes were registered in 30.8% of patients (8/26) and 7.7% of patients (2/26), respectively. According to laparoscopic surgery, POI was combined with pelvic adhesions in 38.5% of cases (10/26). The latter were detected more often than in the comparison group. In addition, 23.1% of patients (6/26) had a history of genitourinary infections, and 38.5% of patients (10/26) had undergone abdominal surgeries that did not directly affect the reproductive organs. 

Transvaginal ultrasonography revealed the presence of residual follicles in the ovaries in 80.8% of patients (21/26), while no follicles were found in 19.2% patients (5/26) at the time of the study. All patients with POI exhibited an extremely diminished ovarian reserve, with AMH level <0.5 ng/mL and antral follicle count (AFC) <5 in both ovaries.

Prior to surgical treatment of POI, 69,2% of patients (18/26) received hormone replacement therapy for 1 (0.25–10) years. The median levels of hormones of the reproductive system, namely AMH, FSH, LH, and estradiol (E2), in the blood serum of patients were 0.025 (0.01–0.4) ng/mL, 22.3 (5–140) mIU/mL, 20.6 (3.13–189) mIU/mL, and 350.6 (26.8–1008) pmol/L, respectively.

Furthermore, many patients with POI had a history of severe infectious diseases, including measles, varicella, and tonsillitis (65.4%), as well as allergic diseases (34.6%). In 34.6% of cases (9/26), a pathology of the thyroid gland was detected, with autoimmune thyroiditis being the most common diagnosis, accompanied by hypofunction and nodular and diffuse tissue changes. However, at the time of examination, the hypofunction of the thyroid gland was compensated by receiving L-thyroxine, and the TSH level was within the reference values in all patients. Moreover, IgG antibodies to TPO and thyroglobulin (TG) were detected in Group 1 in 26.9% (7 of 26) and 7.7% (2 of 26) of patients with POI, respectively, whereas in Group 2 these antibodies were not found. When comparing the two groups, the *p* values were 0.018 and 0.234, respectively. The comparison group comprised women with normal ovarian reserve and a regular menstrual cycle, free from POI and proliferative gynecological diseases (endometriosis, uterine fibroids) according to transvaginal ultrasound, laparoscopic surgery, and hysteroscopy.

ELISA was applied to detect serum autoantibodies against steroidogenic enzymes (CYP11A1, CYP19A1, and CYP21A2) in patients with and without POI (Table 2). This analysis was performed prior to surgery treatment for POI. Patients with POI presented significantly higher median levels of IgG antibodies to steroidogenic enzymes in comparison to patients without POI (*p* < 0.001). In addition, 23.1% of POI patients (6 of 26) had high levels of IgG antibodies against CYP11A1 and CYP19A1 only, but not against CYP21A2.

The level of IgG antibodies to CYP11A1 and CYP19A1 in patients with amenorrhea was 0.411 (0.188; 1.423) OD units and 0.573 (0.283; 1.081) OD units, respectively. This was significantly higher than in patients with oligomenorrhea, who showed levels of 0.284 (0.222; 0.637) OD units and 0.353 (0.226; 0.705) OD units, respectively (*p* values of 0.0259 and 0.047, respectively). The level of these antibodies in the latter was higher than in the comparison group (*p* values of 0.0034 and 0.062, respectively). The levels of IgG antibodies to CYP21A2 in patients with amenorrhea and oligomenorrhea were 0.345 (0.202; 1.184) OD units and 0.330 (0.194; 0.426) OD units, respectively, and did not differ (*p* = 0.461), but in both cases they were higher than in the comparison group (*p* values of 0.0029 and 0.0092, respectively). 

In addition, the Spearman’s rank correlation coefficients (rho) between the levels of IgG antibodies to CYP11A1, CYP19A1, and CYP21A2 and menstrual irregularities in women, including normal menstrual cycle (rank 1), oligomenorrhea (rank 2), and amenorrhea (rank 3), were 0.738, 0.653, and 0.57, respectively (*p* < 0.0001), and were not significantly different (*p* > 0.05). 

The Spearman’s rank correlation coefficients (rho) between the levels of IgG antibodies to CYP11A1, CYP19A1, and CYP21A2 and the antral follicle count according to transvaginal ultrasound examination, including the absence of antral follicles (rank 1), AFC < 5 (rank 2), and AFC ≥ 5 (rank 3), were −0.668, −0.624, and −0.601, respectively (*p* < 0.0001), and also did not differ significantly (*p* > 0.05).

Comparing the levels of serum antibodies to steroid and gonadotropic hormones in the study groups showed that the median levels of IgG antibodies to PG and E2 were significantly higher in patients with POI than in the comparison group (*p* < 0.05) (Table 2). Moreover, the levels of IgG antibodies to FSH and IgM antibodies to all hormones did not differ between the two groups (*p* > 0.05).

In all study groups, serum levels of anti-ovarian IgG antibodies did not increase above the upper limit of the normal range (10 U/mL), and the median levels of anti-ovarian antibodies did not differ between groups (*p* = 0.877) (Table 2).

**Figure 1:**
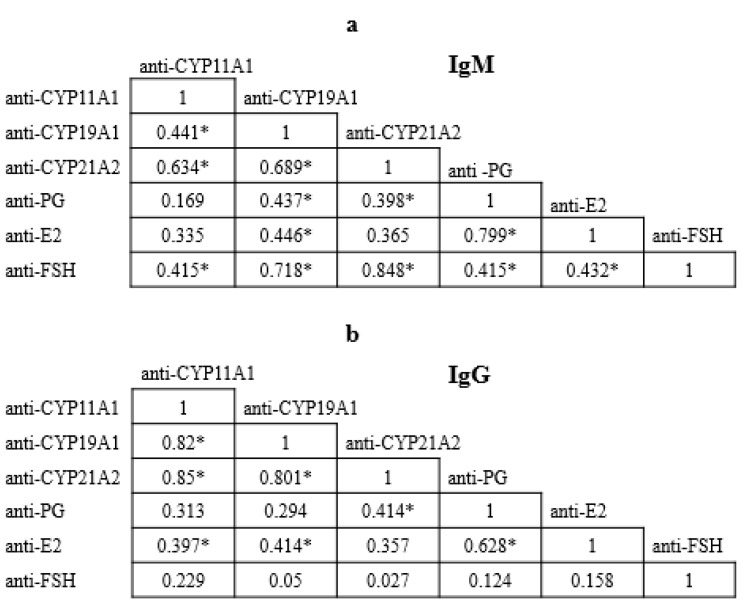
Correlation between the levels of serum IgM (**a**) and IgG (**b**) autoantibodies against steroidogenic enzymes (anti-CYP11A1, anti-CYP19A1, and anti-CYP21A2), estradiol (anti-E2), progesterone (anti-PG), and follicle-stimulating hormone (anti-FSH) in patients with premature ovarian insufficiency. *—*p* < 0.05.

In patients with POI, a direct correlation was found between serum levels of IgG antibodies to the enzymes CYP11A1, CYP19A1, and CYP21A2 (r > 0.8; *p* < 0.0001) (Figure 1) and between IgG antibodies against E2 and PG (r = 0.628; *p* = 0.0006), IgG antibodies against E2 and CYP11A1 or CYP19A1, and IgG antibodies against PG and CYP21A2 (0.397 ≤ r ≤ 0.414; *p* < 0.05). A direct correlation was found between IgM antibodies to FSH and CYP21A2 or CYP19A1 (r values of 0.848 and 0.718, respectively; *p* < 0.0001), IgM antibodies to E2 and PG (r = 0.799; *p* < 0.0001), and IgM antibodies to CYP21A2 and CYP11A1 or CYP19A1 (r values of 0.634 and 0.689, respectively; *p* < 0.001). A direct correlation was also found between IgM antibodies to FSH and E2 or PG, between IgM to PG and CYP21A2, and between IgM to CYP11A1 and CYP19A1 (0.398 ≤ r ≤ 0.441; *p* < 0.05).

According to the receiver operating characteristic (ROC) analysis, IgG antibodies against CYP11A1, CYP19A1, and CYP21A2 had a high diagnostic value for POI, which was confirmed by high sensitivity (76.9%, 65.4%, and 73.1%, respectively), specificity (88.9%, 88.9%, and 83.3%, respectively), and area under the ROC curve (AUC 0.910, 0.850, and 0.842, respectively; *p* < 0.001) (Table 3). Tests for IgG antibodies against E2 and PG showed lower sensitivity (53.9% and 38.5%, respectively) and AUC (0.686 and 0.697, respectively) values for POI.

According to logistic regression analysis, the diagnostic accuracy of tests for IgG antibodies against steroidogenic enzymes reached 75–79.6% for POI. When taking into account the results of testing a panel of three antibodies, the AUC value increased to 0.921 [0.799; 0.981] (*p* < 0.0001), and the diagnostic accuracy reached 84.1%. According to the ROC analysis, in patients with POI, the odds of a positive result in tests for IgG antibodies to steroidogenic enzymes CYP11A1, CYP19A1, and CYP21A2 were 26.8, 14.7, and 13.6 times higher than in women without POI, respectively. At the same time, in patients seropositive for IgG antibodies to the steroidogenic enzymes CYP11A1, CYP19A1, and CYP21A2, the odds of POI were 26.8, 14.7, and 13.6 times higher than in women seronegative for these antibodies, respectively.

A subsequent study of serum autoantibodies performed by ELISA in 15 patients with POI 1–2 months after surgical treatment did not reveal a significant change in the level of autoantibodies of classes M and G (Table 4). The levels of autoantibodies against steroidogenic enzymes and hormones in POI patients did not increase after surgical treatment compared with the levels of the same autoantibodies prior to surgery.

## 3. Discussion

The present pilot study examined the profile of serum autoantibodies, including antibodies against steroidogenic enzymes, steroid and gonadotropic hormones, and thyroid antigens, in patients of reproductive age who meet the international diagnostic criteria for POI with extremely reduced ovarian reserve (AMH < 0.05 ng/mL, AFC < 5 in both ovaries) and with infertility in comparison with a group of women without POI with normal ovarian reserve (AMH level ≥ 1.2 ng/mL, AFC ≥ 5 in both ovaries). 

The study group comprised women aged between 18 and 42 years old with a normal karyotype who were diagnosed with POI in accordance with the ESHRE criteria. The diagnosis was based on the results of clinical, laboratory, and transvaginal ultrasonography examinations. Using laparoscopic visualization, hysteroscopy, and histological examination, proliferative gynecological and oncological diseases were excluded in patients of the study group. Women with iatrogenic forms of POI, systemic autoimmune diseases, and contraindications to surgical treatment, IVF, and pregnancy programs were not included in the study. The analysis of family history showed that the mothers of some patients with POI (in 15.4% of cases) had an earlier onset of menopause (before 45 years of age). According to the findings of Silvén H. et al., having a relative with POI is a remarkable risk factor for POI [5]. Moreover, among relatives of patients with POI, the likelihood of detecting the disease is higher than in the female population (2% vs. 0.5%).

It should be noted that all patients with POI experienced infertility, with primary infertility prevailing over secondary. Adhesive processes in the pelvis were often observed, caused by previous infectious and inflammatory processes, which could contribute to the development of POI and infertility. In most cases, patients had amenorrhea (69.2%), and only in 30.8% of cases women had spontaneous irregular menstrual cycles. However, since the patients with amenorrhea were receiving hormone replacement therapy, only an insignificant decrease or normal level of estradiol and an increase of varying degrees in FSH levels were observed in Group 1 at the time of the study. 

The most prevalent somatic pathology was autoimmune thyroiditis with thyroid hypofunction and an increased level of antibodies to TPO (in 26.9% of cases). At the time of examination, hypofunction of the thyroid gland was compensated in all patients by receiving L-thyroxine. The results obtained are consistent with the data of a recent study, which also showed a significant increase in the level of antithyroid antibodies in patients with POI (in 37.9% of cases) compared with healthy women [21]. According to 30 studies, women of reproductive age with Hashimoto’s thyroiditis are characterized by a decrease in AMH level and antral follicle count [22]. A retrospective cohort study showed an 89% higher risk of amenorrhea in patients with Hashimoto’s thyroiditis than in patients without autoimmune thyroid disease [23].

It is important to note that the comparison group consisted of women of a similar age as in the main group, with normal ovarian reserve, regular menstrual cycle, and normal thyroid function, and without proliferative gynecological diseases according to laparoscopic surgery, hysteroscopy, and histological examination data.

As is well known, POI is a polyetiological disease, which is often associated with the involvement of various factors. In the present study, a significant proportion of patients (34.6%) associated the onset of POI with the effect of previous stress factors. Although the link between long-term psychological stress and ovarian function is clear, few studies have shown an association between POI and stress, and further research is required to understand the mechanisms of stress-induced dysfunction [24]. 

The immune system is known to play a critical role in ovarian pathophysiology [1]. In addition, systemic pro-inflammatory conditions may have a negative impact on follicular dynamics, leading to changes in ovarian homeostasis [25]. In this regard, it is important to note that patients with POI in the study group exhibited a high incidence of infectious and allergic diseases, which could be attributed to immune dysfunction, and the latter, in turn, could predispose to the development of an autoimmune form of POI. 

Furthermore, it is notable that patients with POI had often had urogenital infections and abdominal surgeries in the past, which could potentially lead to inflammatory processes involving the reproductive organs, particularly the ovaries. The presence of pelvic adhesions in a significant proportion of patients (38.5%) confirm this assumption. Autoimmune lymphocytic oophoritis frequently precedes the onset of POI. It is associated with the production of autoantibodies to steroidogenic enzymes and, according to histological data, manifests itself in the form of mononuclear inflammatory infiltrates in the theca cells of preantral and antral follicles [15]. Apparently, the described cellular and humoral immune reactions affect steroid-producing cells and block the production of androstenedione and estradiol. Additionally, these reactions result in the damage and destruction of growing theca cells and follicles, as well as the degeneration and atrophy of the ovaries, ultimately leading to POI.

However, in a majority of cases, the leading etiological factor cannot be identified due to a number of reasons, in particular due to the insufficient effectiveness of diagnostic methods and the late visits of patients to the doctor because of the mild severity of symptoms at the early stage of the disease and the insufficient awareness of patients. At the last stage of POI, ovarian atrophy develops, while histological examination reveals fibrosis and the absence of ovulation stigmas [26]. In such cases, hormone replacement therapy and IVF programs with donor eggs are offered as potential solutions, although some patients decline the latter option for ethical or social reasons. In this regard, the primary objectives remain the early diagnosis of POI before the manifestation of clinical symptoms, the identification of the etiology of the disease, and an individual approach to complex treatment.

In the present study, despite the late stage of POI, residual follicles in the ovaries were detected by transvaginal ultrasonography in most patients. However, previous conservative treatment and IVF attempts, both with the patient’s own and donor oocytes, were ineffective in relation to the implementation of a woman’s reproductive function.

The ineffectiveness of conservative therapy and IVF programs in patients indicated the necessity for surgical treatment of POI. To realize the reproductive function of women using their own oocytes, new methods of reproductive surgery were employed. Previously, Kawamura K. et al. demonstrated that Hippo and Akt signaling pathways regulate follicle growth and first proposed a surgical method for the in vitro activation of residual follicles in infertile patients to disrupt Hippo signaling and drug-stimulate Akt signaling [27]. Adamyan L.V. et al. developed the single-stage surgical procedure for the treatment of POI and the recovery of ovarian function [28]. Complex treatment of POI, including new surgical technology and a subsequent IVF program, was successful and led to pregnancy in 22 (24.7%) patients, including 16 (17.9%) patients using their own oocytes [29]. 

It is important to note that, to date, specific autoimmune markers of POI have not been identified for diagnosing the early stages and predicting the development of the disease. Consequently, the present study examined a wide spectrum of serum autoantibodies in patients with POI, including autoantibodies to steroidogenic enzymes, steroid and gonadotropic hormones, and thyroid antigens, by means of modified ELISAs using human recombinant enzymes (CYP11A1, CYP19A1, and CYP21A2). The most significant steroidogenic enzymes of cytochrome P450, which are expressed in the ovaries and other organs and tissues, were selected as targets, namely the cholesterol side-chain cleavage enzyme (P450scc, CYP11A1) and aromatase (P450arom, CYP19A1) [30]. As is known, these enzymes catalyze the first reaction in the biosynthesis of all steroid hormones and the last reaction in the biosynthesis of estrogen, respectively. The adrenal cortex 21-hydroxylase (P450c21, CYP21A2), which is involved in the biosynthesis of aldosterone and cortisol, was also used as a target.

The profile of serum autoantibodies in patients with POI was characterized by increased levels of IgG antibodies against steroidogenic enzymes (CYP11A1, CYP19A1, and CYP21A2) and steroid hormones (PG, E2) compared to women without POI. At the same time, the levels of anti-ovarian antibodies and IgM antibodies to enzymes and hormones did not differ between the two groups. 

It is important to note that 23.1% of patients (6 out of 26) exhibited an increase in the level of IgG antibodies to CYP11A1 and CYP 19A1 without any increase in the level of antibodies to CYP21A2. Apparently, antibodies to enzymes (CYP11A1 and CYP 19A1) contained in the ovaries are more specific for POI than antibodies to an enzyme (CYP21A2) contained in the adrenal cortex.

In Group 1, the levels of IgG antibodies to CYP11A1, CYP19A1, and CYP21A2 were found to be significantly higher not only in women with amenorrhea but also in women with oligomenorrhea, i.e., at an earlier stage of POI, compared to Group 2. At the same time, in patients with amenorrhea, the level of antibodies to CYP11A1 and CYP19A1 was higher than in patients with oligomenorrhea. A significant direct correlation was found between the levels of IgG antibodies against CYP11A1, CYP19A1, and CYP21A2 and menstrual irregularity in women (rho 0.738, 0.653, and 0.57, respectively). Moreover, an inverse correlation was observed between the levels of IgG antibodies to CYP11A1, CYP19A1, and CYP21A2 and the number of antral follicles in the ovaries (rho −0.668, −0.624, and −0.601, respectively). In both cases, higher correlation coefficients were obtained for antibodies to CYP11A1 and CYP19A1. The results obtained suggest the involvement of these autoantibodies in the pathogenetic mechanisms of POI, such as blocking estrogen biosynthesis and damage and destruction of follicles in the ovaries. Therefore, oocyte cryopreservation for future IVF programs may be offered to women with high levels of these antibodies and fertility problems.

In addition, a direct correlation has been found between antibodies (M, G) against E2, IgM to FSH, and antibodies to CYP11A1 or CYP19A1, which are present in the ovaries and provide estrogen biosynthesis. Antibodies to CYP11A1 and CYP19A1 appear to be more specific for POI and correlate with ovarian dysfunction. A direct correlation was also found between the levels of IgG antibodies to PG and antibodies to CYP21A2. The latter mediates the conversion of progesterone and 17-hydroxyprogesterone in the early stages of the biosynthesis of aldosterone and cortisol, respectively, in the adrenal cortex, and antibodies to CYP21A2 are more specific for autoimmune Addison’s disease.

The present study demonstrated the high diagnostic value of IgG antibodies against CYP11A1, CYP19A1, and CYP21A2 for POI (AUC values 0.842–0.910), as well as the high diagnostic accuracy of these tests (75–79.6%). The highest values of sensitivity (76.9%), specificity (88.9%), and AUC (0.910 [0.785; 0.975]) were obtained for IgG antibodies to CYP11A1. It is important to note that the determination of antibodies to CYP11A1 and CYP19A1 in addition to the antibodies to CYP21A2 recommended by the ESHRE allowed us to identify a larger number of seropositive patients with POI (23.1% more). Moreover, when determining a panel of these antibodies, an increase in AUC to 0.921 and diagnostic accuracy to 84.1% was observed. These results confirm the usefulness of using antibodies to the CYP11A1 and CYP19A1 enzymes contained in the ovaries as additional specific markers for the diagnosis of POI.

Note that the production of autoantibodies in POI patients 1–2 months after surgical treatment remained at the same level as prior to surgery. The results of the present study showed that the use of new surgical technologies for the treatment of POI is harmless in terms of triggering the production and increasing the level of autoantibodies in POI patients.

The results of this study are consistent with the data of Vogt et al., who, using a radiolabeled ligand immunoprecipitation method, showed a high prevalence of antibodies against P450scc in POI in patients with Addison’s disease [31]. The determination of these antibodies was characterized by high sensitivity (72%) and specificity (84%) for POI, comparable to those obtained by us in the present study using a modified ELISA. Moreover, the anti-P450scc antibody test is more specific for POI and can be used to diagnose POI in patients with autoimmune Addison’s disease who already have antibodies to 21OH. Testing for autoantibodies against P450scc is recommended for all young women under 40 years of age with menstrual irregularities or fertility problems in cases of autoimmune primary adrenal insufficiency [32].

The production of antibodies to steroid hormones could be associated with the long-term use of hormone replacement therapy by patients, whereas a compensatory increase in the level of gonadotropins, particularly FSH, in patients with POI could contribute to the production of autoantibodies to FSH. It is assumed that antibodies found in association with POI, directed against the FSH β-subunit, can reduce the biological activity of the hormone and affect ovarian function by modulating the recognition and binding of FSH to its receptor, which is consistent with the data of other authors [33].

Due to a better understanding of the role of the immune system in the pathophysiology of autoimmune POI, it is important to create and test new treatments for this condition [8]. Additional randomized controlled trials with larger sample sizes are needed to evaluate the effectiveness of existing and proposed new treatments.

Thus, effective treatment of POI requires early diagnosis with determination of the autoantibody profile and a personalized approach to complex treatment, including: hormonal replacement and antioxidant therapy; the correction of autoimmune disorders using immunosuppressive, efferent, and immune therapy; intra-ovarian injections (stem cells and platelet-rich plasma); surgical treatment; and IVF programs.

The limitations of this study were the small size of the study and comparison groups, restricted by the indications for surgical treatment of patients with POI using new surgical technologies and the exclusion of patients with proliferative gynecological and oncological diseases, the presence of other gynecological and somatic diseases in patients of the comparison group, and the use of homemade ELISA tests and controls due to a lack of commercial tests and standards. Moreover, not all endogenous mechanisms triggering autoantibody production and not all possible confounders were taken into account in this study.

## 4. Materials and Methods

### 4.1. Ethics and Sample Collection

The project of this study was approved by the Ethics Committee of the National Medical Research Center for Obstetrics, Gynecology and Perinatology named after Academician V.I. Kulakov. Patients signed voluntary informed consent to participate in the study and for their biological material to be tested. All patients underwent laparoscopic surgery and hysteroscopy in the gynecological department of the Department of Operative Gynecology and General Surgery of the National Medical Research Center for Obstetrics, Gynecology and Perinatology named after Academician V.I. Kulakov between November 2022 and December 2023.

The main group (Group 1) comprised 26 patients aged 18–42 years with POI and extremely reduced ovarian reserve (AMH level < 0.5 ng/mL and antral follicle count (AFC) < 5 in both ovaries, according to transvaginal ultrasonography) and normal karyotype and number of CGG repeats in the FMR-1 gene. POI was diagnosed in patients according to the ESHRE criteria. POI patients underwent laparoscopic surgery hysteroscopy, biopsy of the ovarian cortex, and subsequent histological examination.

Patients with proliferative gynecological diseases (endometriosis or uterine fibroids), oncological and systemic autoimmune diseases, as well as contraindications for surgical treatment, IVF programs, and pregnancy, were excluded from the study.

The comparison group (Group 2) consisted of 18 women of reproductive age with normal ovarian reserve (AMH level ≥ 1.2 ng/mL, FSH < 12 mIU/mL, and AFC ≥ 5 in both ovaries) and without POI and proliferative gynecological diseases, which was confirmed by transvaginal ultrasonography, laparoscopic surgery, and hysteroscopy.

To assess the profile of serum autoantibodies, blood samples were taken from fasting women in the follicular phase of the menstrual cycle on day 11.9 ± 4.2 immediately prior to laparoscopic surgery, as well as 1–2 months after surgical treatment. Serum samples were processed according to standard methods, including centrifugation at 3000 rpm for 10 min at a temperature of 4 °C, and stored at −80 °C in the centralized biobank of the National Medical Research Center for Obstetrics, Gynecology and Perinatology named after Academician V.I. Kulakov for 1 year before testing. The entire collection of serum samples was tested simultaneously and was not subjected to repeated freeze–thaw cycles.

### 4.2. ELISA for Detection of Serum Autoantibodies

Serum autoantibodies to steroidogenic cytochrome P450 enzymes were determined by means of a modified version of the indirect enzyme-linked immunosorbent assay (ELISA) described previously [34], using human recombinant enzymes CYP21A2 (Asp106~Gln389), CYP11A1 (Val392~Gln521), and CYP19A1 (Leu196~Val373) expressed in E. coli (Cloud-Clone Corp., Katy, TX, USA) as the target, Thermo Scientific™ Nunc™ MaxiSorp microplates (Thermo Fisher Scientific Inc., Waltham, MA, USA), and horseradish peroxidase–labeled monoclonal antibodies against human IgM and IgG (XEMA LLC, Moscow, Russia), reagents and buffers for ELISA (XEMA LLC, Moscow, Russia).

Human recombinant enzymes CYP21A2, CYP11A1, or CYP19A1 were adsorbed onto polystyrene 96-well microplates with a high protein binding capacity in an amount of 125 ng per well in 100 mM phosphate-buffered saline (PBS), pH 7.4, by incubation for 16 h at 20 ± 2 °C, instead of incubating 10 ng of recombinant protein per well in 50 mM carbonate/bicarbonate buffer (pH 9.6) at 4 °C overnight. Nonspecific binding sites were blocked with 1% (weight/volume [*w*/*v*]) bovine serum albumin (BSA) in PBS by incubating for 1.5 h at room temperature (20 ± 2 °C). The blood serum samples were analyzed at a 1:100 dilution in 0.5% (*w*/*v*) BSA-PBS containing 0.05% (*v*/*v*) Tween-20 in the duplicate, incubated at room temperature on a shaker at 200 rpm for 1 h. Conjugates against human IgM and IgG were incubated in working dilutions in BSA-PBS under the same conditions. 3,3′,5,5′-tetramethylbenzidine (TMB) was used as a substrate instead of 2,2′-azino-bis-(3-ethylbenzothiazoline-6-sulphonic acid) (ABTS), and the absorbance of the solution was measured at 450 nm instead of 415 nm. In each assay, positive and negative control sera were analyzed along with the test samples. Optical density (OD) was measured on an Infinite F50 automatic enzyme immunoassay analyzer (TECAN Austria GmbH, Grödig, Austria) at a wavelength of 450 nm. The intra- and inter-assay coefficients of variation in these ELISAs were <10% and <15%, respectively.

Antibodies (M, G) to steroid hormones, namely progesterone (PG) and estradiol (E2), were detected by means of modified ELISA as previously described [35], using Thermo Scientific™ Nunc™ MaxiSorp microplates (Thermo Fisher Scientific Inc., Waltham, MA, USA), β-estradiol 6-(O-carboxymethyl)oxime: BSA conjugate (Sigma-Aldrich, Burlington, VT, USA), and progesterone 3-(O-carboxymethyl)oxime: BSA conjugate (XEMA LLC, Moscow, Russia). 

Antibodies (M, G) to follicle-stimulating hormone (FSH) were detected by means of a modified ELISA using human FSH preparation (Sigma-Aldrich, Burlington, VA, USA), as previously described [36]. 

Detection of anti-ovarian antibodies (AOA) class G was performed using immunoenzyme kits (DRG Instruments GmbH, Marburg, Germany) for the quantitative measurement of antibodies directed against oocytes in human serum.

### 4.3. Statistical Analysis

Statistical analysis of the results was performed using application packages Microsoft Office Excel 2016 (Microsoft Corp., Redmond, Washington, DC, USA) and Statistics 10 (StatSoft Inc., Tulsa, OK, USA). The normality of the distribution of values in the samples was determined using the Shapiro–Wilk and Kolmogorov–Smirnov tests. Quantitative data were presented as the arithmetic mean and standard deviation (M ± SD) in the case of a normal distribution or as the median (Me) and the range of values from minimum (min) to maximum (max) (Me (min; max)) in case of deviation from normal distribution. Qualitative data were presented as absolute (n) and relative values (%), and the presence of differences between values in groups was determined using the χ2 test. The *t*-test or Mann–Whitney U test was used to assess differences between continuous variables. The presence of a relationship between quantitative variables was assessed by calculating the Spearman correlation coefficient (r). To assess the relationship between a quantitative variable and a qualitative characteristic, the Spearman rank correlation coefficient (rho) was calculated. The association between risk factors and outcome was assessed by calculating the odds ratio (OR). Receiver operating characteristic (ROC) analysis was performed to assess the relationship between the independent variables and the dependent binary variable. The diagnostic accuracy of the tests was estimated using logistic regression analysis. Differences were considered statistically significant at a significance level of *p* < 0.05.

## 5. Conclusions

The results of this study shown that POI is associated with the presence of serum autoantibodies against steroidogenic enzymes, steroid hormones (estradiol and progesterone), and thyroid peroxidase. Autoantibodies against CYP11A1, CYP19A1, and CYP 21A2 are risk factors for POI and, according to preliminary data, have a high diagnostic value for POI. The levels of these antibodies correlate with menstrual irregularities and a decrease in the antral follicle count in the ovaries. Screening for antibodies against CYP11A1 and CYP19A1 in addition to antibodies against CYP21A2 may improve the accuracy of the diagnosis of autoimmune POI and be useful for detecting early stages and predicting POI progression, as well as identification of the high-risk groups for POI among women with menstrual irregularities and fertility problems.

We believe that it is necessary to conduct further studies involving a larger group of patients with POI and a control group of women to detail the findings, to establish standardized effective tests for the detection of autoantibodies against steroidogenic enzymes for routine laboratory practice, to assess the effectiveness of using autoimmune markers for early diagnosis of POI and predicting disease progression, to determine clinical and laboratory criteria for inclusion in the POI high-risk groups, and to find new, effective methods for the prevention and treatment of POI.

## Figures and Tables

**Table 1:** Clinical, anamnestic, and demographic characteristic of patients with POI and women without POI.

Parameter	POI (Group 1, n = 26)	Non-POI (Group 2, n = 18)	*p* Value
Age, years	35.2 ± 4.87	32.3 ± 5.75	0.078 *
BMI, kg/m^2^	21 (17–38)	21 (17–32)	0.72 **
BMI > 25 kg/m^2^	6 (23.1%)	3 (16.7%)	0.609 ***
Amenorrhea	18 (69.2%)	0	<0.0001 ***
Oligomenorrhea	8 (30.8%)	0	0.01 ***
Gravidity	0 (0–3)	0 (0–6)	0.0046 **
Parity	0 (0–1)	0 (0–5)	0.793 **
Primary infertility	17 (65.4%)	4 (22.2%)	0.0053 ***
Secondary infertility	9 (34.6%)	5 (27.8%)	0.638 ***
Duration of infertility, years	3 (1–20)	2 (1–5)	0.041 **
In vitro fertilization	10 (38.5%)	4 (22.2%)	0.259 ***
Pelvic adhesions	10 (38.5%)	2 (11.1%)	0.047 ***
Genitourinary infections	6 (23.1%)	2 (11.1%)	0.316 ***
Abdominal surgery	10 (38.5%)	2 (11.1%)	0.047 ***
Thyroid diseases	9 (34.6%)	0	0.0057 ***
Infectious diseases	17 (65.4%)	6 (33.3%)	0.0383 ***
Allergic diseases	9 (34.6%)	3 (16.7%)	0.0764 ***

Note: *—M ± δ, *t*-test; **—Me (min; max), Mann–Whitney U test; ***—n (%), ꭓ2-test. Abbreviation: POI, premature ovarian insufficiency; BMI, body mass index.

**Table 2:** Level of serum autoantibodies against steroidogenic enzymes (CYP21A2, CYP11A1, and CYP19A1), steroid and gonadotropic hormones (OD units), and anti-ovarian antibodies (U/mL) in patients with POI and women without POI.

Parameter	POI (Group 1, n = 26)	Non-POI (Group 2, n = 18)	*p* Value *
Anti-CYP11A1 IgM	0.211 (0.162; 0.296)	0.224 (0.123; 0.374)	0.535
Anti-CYP11A1 IgG	0.372 (0.188; 1.423)	0.200 (0.131; 0.337)	<0.0001
Anti-CYP19A1 IgM	0.188 (0.093; 0.433)	0.179 (0.106; 0.322)	0.905
Anti-CYP19A1 IgG	0.471 (0.226; 1.08)	0.247 (0.100; 0.579)	0.0001
Anti-CYP21A2 IgM	0.282 (0.183; 0.482)	0.297 (0.201; 0.368)	0.703
Anti-CYP21A2 IgG	0.344 (0.194; 1.184)	0.225 (0.162; 0.394)	0.0001
Anti-PG IgM	0.199 (0.092; 0.737)	0.207 (0.119; 0.280)	0.756
Anti-PG IgG	0.203 (0.110; 0.497)	0.159 (0.098; 0.247)	0.028
Anti-E2 IgM	0.210 (0.102; 1.016)	0.187 (0.101; 0.312)	0.650
Anti-E2 IgG	0.313 (0.162; 1.045)	0.257 (0.141; 0.401)	0.038
Anti-FSH IgM	0.256 (0.196; 0.519)	0.264 (0.149; 0.400)	0.667
Anti-FSH IgG	0.222 (0.125; 0.526)	0.227 (0.114; 0.477)	0.583
AOA IgG	2.85 (1.88; 6.56)	2.88 (1.78; 6.30)	0.877

Note: *—Me (min; max), Mann–Whitney U test. Abbreviation: POI, premature ovarian insufficiency; CYP11A1, cholesterol side-chain cleavage enzyme; CYP19A1, aromatase; CYP21A2, 21-hydroxylase; PG, progesterone; E2, estradiol; FSH, follicle-stimulating hormone; AOA, anti-ovarian antibodies.

**Table 3:** Diagnostic value of serum autoantibodies against steroidogenic enzymes (CYP21A2, CYP11A1, and CYP19A1) and steroid hormones for premature ovarian insufficiency according to the ROC analysis.

Parameter	Cutoff (OD Units)	Se *, %	Sp, %	AUC (95% CI)	Accuracy ** (%)	DOR	*p* Value
Anti-CYP11A1 IgG	0.286	76.9	88.9	0.910 [0.785; 0.975]	79.6	26.7	0.0002
Anti-CYP19A1 IgG	0.362	65.4	88.9	0.850 [0.711; 0.940]	75	15.1	0.0015
Anti-CYP21A2 IgG	0.275	73.1	83.3	0.842 [0.700; 0.934]	75	13.6	<0.001
Anti-E2 IgG	0.303	53.9	83.3	0.686 [0.528; 0.817]	61.4	5.83	0.0179
Anti-PG IgG	0.229	38.5	83.3	0.697 [0.540; 0.826]	63.6	3.12	0.129

Note: *—maximum sensitivity at specificity of >80%; **— (true-positive cases + true-negative cases)/total number of cases × 100. Abbreviation: ROC, receiver operating characteristic; Se, sensitivity; Sp, specificity; AUC, area under the ROC curve; CI, confidence interval; DOR, diagnostic odds ratio; CYP11A1, cholesterol side-chain cleavage enzyme; CYP19A1, aromatase; CYP21A2, 21-hydroxylase; PG, progesterone; E2, estradiol.

**Table 4:** Levels of serum autoantibodies against steroidogenic enzymes (CYP21A2, CYP11A1, and CYP19A1) and steroid and gonadotropic hormones (OD units) in patients with POI (n = 15) prior to surgery and 1–2 months after surgery.

Parameter	Prior to Surgery	After Surgery	*p* Value *
Anti-CYP11A1 IgG	0.337 (0.188–1.423)	0.324 (0.220–1.500)	0.787
Anti-CYP11A1 IgM	0.176 (0.162–0.272)	0.201 (0.154–0.283)	0.576
Anti-CYP19A1 IgG	0.420 (0.249–1.08)	0.426 (0.144–1.103)	0.604
Anti-CYP19A1 IgM	0.162 (0.116–0.433)	0.176 (0.087–0.359)	0.852
Anti-CYP21A2 IgG	0.308 (0.202–1.184)	0.270 (0.189–1.231)	0.254
Anti-CYP21A2 IgM	0.235 (0.183–0.482)	0.263 (0.168–0.560)	0.683
Anti-PG IgG	0.203 (0.110–0.408)	0.189 (0.119–0.249)	0.329
Anti-PG IgM	0.195 (0.092–0.391)	0.169 (0.109–0.440)	0.984
Anti-E2 IgG	0.295 (0.178–0.591)	0.277 (0.205–0.507)	0.576
Anti-E2 IgM	0.144 (0.102–0.440)	0.166 (0.103–0.473)	0.548
Anti-FSH IgG	0.239 (0.125–0.526)	0.232 (0.115–0.532)	0.604
Anti-FSH IgM	0.235 (0.183–0.482)	0.263 (0.168–0.560)	0.683

Note: *—Me (min; max), Mann–Whitney U test. Abbreviation: POI, premature ovarian insufficiency; CYP11A1, cholesterol side-chain cleavage enzyme; CYP19A1, aromatase; CYP21A2, 21-hydroxylase; PG, progesterone; E2, estradiol; FSH, follicle-stimulating hormone.

## Data Availability

The data that support the findings of this study are available from the corresponding author upon request.
